# Genome Sequence of *Arenibacter algicola* Strain SMS7, Found in Association with the Marine Diatom *Skeletonema marinoi*

**DOI:** 10.1128/MRA.01461-18

**Published:** 2019-01-10

**Authors:** Mats Töpel, Matthew I. M. Pinder, Oskar N. Johansson, Olga Kourtchenko, Anna Godhe, Adrian K. Clarke

**Affiliations:** a Department of Marine Sciences, University of Gothenburg, Gothenburg, Sweden; b Gothenburg Global Biodiversity Centre, Gothenburg, Sweden; c Department of Biological and Environmental Sciences, University of Gothenburg, Gothenburg, Sweden; University of Maryland School of Medicine

## Abstract

Arenibacter algicola strain SMS7 was isolated from a culture of the marine diatom Skeletonema marinoi strain ST54, sampled from top-layer sediments in Kosterfjord, Sweden. Here, we present its 5,857,781-bp genome, consisting of a circular chromosome and one circular plasmid, in all containing 4,932 coding sequences.

## ANNOUNCEMENT

In an ongoing study of the microbiome of the chain-forming diatom Skeletonema marinoi strain ST54, we isolated and sequenced the associated bacterial strain SMS7.

The ST54 culture was established from a revived resting cell taken from top-layer sediment in Kosterfjord, Sweden (58**°**51.0′N, 10**°**45.7′E; 102 m depth) in May 2009. This bacterial strain was sampled from a colony formed after multiple-dilution streaking on marine agar. Genomic DNA was extracted using Plant DNAzol reagent (Invitrogen Life Technologies, USA) from pure cultures grown from a single bacterial colony, according to the manufacturer’s instructions. Genome sequencing was performed with the PacBio RS II platform (Pacific Biosciences, Menlo Park, CA, USA) on a single-molecule real-time (SMRT) cell. The sequencing produced 98,352 uncorrected reads totaling 1.3 Gbp, which were assembled using Falcon version 1.7.5 (https://github.com/PacificBiosciences/FALCON [1]; seed read length, 17,000 bp). To ensure that the contigs were circular, the corresponding contig ends were joined, and the SMRT Portal version 2.3.0 RS_Resequencing.1 protocol (Pacific Biosciences [2]) was used to remap the reads to the contigs; this included a correction step using Quiver (2). The assembly contains two circular contigs, a chromosome of 5,793,053 bp (G+C content, 39.8%) and a plasmid of 64,728 bp (G+C content, 43.8%), with an average assembly read coverage of 173.06× (statistics are summarized in Table 1).

**TABLE 1:** Summary of assembly and annotation statistics for *Arenibacter algicola* strain SMS7

	Value for:		
Statistic	Total assembly	Chromosome	pSMS7
Assembly			
No. of reads	98,352		
No. of bases	1,266,330,556		
Final assembly size (bp)	5,857,781	5,793,053	64,728
G+C content (%)	39.8	39.8	43.8
Avg read coverage (×)	173.06		
Annotation (no.)			
CDSs	4,932	4,852	80
Pseudogenes	17	17	0
tRNAs	47	47	0
rRNAs	9	9	0
ncRNAs	22	22	0
tmRNAs	1	1	0

The assembly was annotated with Prokka version 1.12beta (3); this inferred 4,932 coding sequences (CDSs; of which 4,061 have a predicted function), 17 pseudogenes, 47 tRNAs, 9 rRNAs, 22 noncoding RNAs (ncRNAs), and one transfer-messenger RNA (tmRNA) (statistics are summarized in Table 1). Strain SMS7’s chromosome contains three identical 16S rRNA sequences, which share 99.9% identity with the three found in the Arenibacter algicola type strain TG409 (NCBI RefSeq accession number NZ_JPOO00000000). In addition, the housekeeping genes *gyrB* and *rpoB* show 99.1% and 99.0% sequence similarity, respectively, between strain SMS7 and A. algicola strain TG409^T^. Given this similarity, strain SMS7 was compared to all whole-genome-sequenced *Flavobacteriaceae* species available in RefSeq (ftp://ftp.ncbi.nlm.nih.gov/genomes/refseq/bacteria/) using the phylotaxonomic analysis software PhyloPhlAn version 0.99 (4). This showed strain SMS7 as sister to the clade of A. algicola TG409^T^ and Arenibacter sp. strain C-21, with 100% bootstrap support. Taking the above-described analyses together, we place strain SMS7 in the taxon Arenibacter algicola. In addition, colonies of strain SMS7 showed the characteristic orange pigmentation of A. algicola, attributed to its pigment absorbing at 450/470/476 nm.

Arenibacter algicola strain SMS7 contains a plasmid, pSMS7 (with 80 predicted CDSs), a feature not reported for strain TG409^T^ (5). This plasmid was compared to the type strain assembly and SMS7 chromosome using BLASTn (6), and the result implies that pSMS7 is a unique replicon, as no sizable equivalent appears in the strain TG409^T^ assembly or the SMS7 chromosome.

The A. algicola type strain TG409 was originally isolated from the Skeletonema type species, S. costatum (7). Our identification of another A. algicola strain associated with S. marinoi provides further evidence of functional links between the two organisms. One suggested link is the diatoms’ ability to accumulate polycyclic aromatic hydrocarbons (PAHs) on their silica frustules, which associated A. algicola bacteria can use as a carbon source (7).

### Data availability.

This whole-genome project has been deposited in GenBank under the accession numbers CP022515 and CP022516 as part of BioProject number PRJNA380207.
